# Managing Early Childhood Caries for Young Children in China

**DOI:** 10.3390/healthcare6010011

**Published:** 2018-01-30

**Authors:** Kitty Jieyi Chen, Sherry Shiqian Gao, Duangporn Duangthip, Edward Chin Man Lo, Chun Hung Chu

**Affiliations:** Faculty of Dentistry, The University of Hong Kong, Hong Kong, China; kjychen@hku.hk (K.J.C.); gao1204@hku.hk (S.S.G.); hrdplcm@hku.hk (E.C.M.L.); chchu@hku.hk (C.H.C.)

**Keywords:** dental caries, tooth, oral health, children, fluoride

## Abstract

The latest national survey found that 70% of 5-year-old children in China had dental caries. The prevalence of early childhood caries (ECC) may not only be attributed to poor oral hygiene and unhealthy diet, but also to limited access to and availability of dental care. The prevailing preventive measures adopted by industrialised countries for ECC management are neither practical nor affordable in China. Hence, an alternative approach to ECC management is necessary. Atraumatic restorative treatment (ART) has been advocated because the simple and short operative time renders ART affordable. However, the success rate of ART in restoring anterior primary teeth is unfavourable. Although there is no water fluoridation in China, topical fluorides may be used to manage ECC. Tooth brushing with fluoride toothpaste is effective for caries control, but not all toothpastes in China are fluoridated. Professionally applied fluorides such as sodium fluoride varnish can be a cost-effective treatment for managing the high prevalence of ECC in China. Silver diamine fluoride (SDF) at 38% is suggested to be effective in arresting ECC in China. It can be a simple, non-invasive and low-cost treatment. However, it stains caries black. Children and their parents must be well informed before SDF treatment.

## 1. Introduction

Early childhood caries (ECC) is the term used to describe the presence of decayed, missing or restored teeth in primary dentition of children younger than 6 years [1]. The World Health Organisation (WHO) considers ECC to be one of the most prevalent diseases in childhood [2]. Back in the 1980s, the WHO set a goal that 50% of 5- to 6-year-old children should be caries-free by the year 2000 [3]. Unfortunately, this goal could not be achieved. A review in 2017 found that caries prevalence and experience were high among preschool children in Southeast Asia [4]. The median caries prevalence was 79% among 5- to 6-year-old children. ECC is also prevalent among young children in China. Among the 7.6 billion people in the world, about 19% (1.4 billion) of them are living in China. The fourth national oral health survey in China conducted in 2015 found that more than two-thirds (70.9%) of 5-year-old children had experienced caries [5]. Compared to the data of the third national oral health survey in 2005, the caries prevalence rate had increased by 5.8% [6]. Within the country, there is a wide geographic variation of ECC prevalence across China [7]. The caries experience of the children in rural areas was higher than those living in urban areas [8]. The caries experiences in the mean dmft (decayed, missing and filled teeth) score of children 5 to 6 years old in urban and rural areas of southern China were 4.8 and 7.0, respectively. Furthermore, over 90% of their decayed primary teeth were unrestored or untreated. Sociodemographic background, oral health related behaviours and dietary habits were associated with ECC prevalence in China [9,10]. Approximately, one in seven brushed their teeth twice a day [11]. Early life behavioural factors, such as breastfeeding and age of children when toothbrushing started, were significantly associated with caries experience in Chinese preschool children [11].

### Dental Care for Young Children in China

The severe problem of ECC among young children in China may be attributed to the poorly developed dental service [12]. The dentist-to-population ratio is 1:10,000, revealing a shortage of dentists in China [13]. In addition, there is a skewed distribution of dental health personnel and services between rural and urban areas, which may account for the poorer access to dental care for children residing in rural areas. Although there has been substantial improvement in dental technology and care in recent years, young children have not benefitted much. In many regions of China, the prevailing preventive schemes proposed by developed countries for caries management are neither affordable nor practical. Furthermore, the efforts to provide dental caries treatment in remote and developing areas such as the use of mobile dental clinics with portable equipment were put forward, but the technology is often too complex for sustained use [14]. The cost of the required dental instruments and materials and infection control protocol is high, and training for dental personnel to promote basic oral care is insufficient. Hence, an alternative approach of ECC management is necessary. This study aimed to discuss the management of ECC using atraumatic restorative treatment (ART) to restore the caries and fluoride agents to arrest the caries of children in China.

## 2. Atraumatic Restorative Treatment

Restorative treatment of primary teeth is important for function, speech, aesthetics and maintaining space for the eruption of permanent teeth. Conventional dental restorations with composite resin and amalgam required complicated clinical procedures and dental units in a clinic setting. ART has been advocated as an alternative treatment in areas where logistic support such as electricity is limited [15]. ART is a minimally invasive and pain-free restorative treatment. Carious dental tissues are excavated by basic hand instruments without drilling or local anaesthetics. Glass ionomer cement is then inserted into the cavity. At the same time, the adjacent pits and fissures can be sealed with glass ionomer cement using finger pressure technique. ART is straightforward because the filling is a single application with no increment. It provides an alternative approach to the management of ECC in particular under a primary dental care setting. It is a more cost-effective method than conventional restorative treatment in treating cavitated ECC in young children. The advantages of glass ionomer cement include its ability to bond to tooth structure, and the fluoride released from the material is important to prevent secondary caries formation and progression. The simple and short operative time of ART renders this approach of alternative care affordable to young children in China. In addition, the basic and simple instruments needed makes ART a popular technique in community dental services. A 30-month trial in China reported ART restorations placed in preschool children showed promising results [16]. The trial found that the retention rate (79%) of the Class I posterior restoration was satisfactory, while the retention rate of Class II posterior restorations was moderate (51%) after 30 months. However, the success rate of Class III and IV ART restorations was very low in that study. Two-thirds of restorations in anterior teeth failed within the first year after ART placement.

A systematic review and meta-analysis found a high survival rate (86%) of ART over 3 years on single-surface caries in primary teeth [17]. However, the survival of multi-surface ART restorations in primary teeth was less satisfactory. An annual failure rate of 17% was reported compared with 5% for single-surface restorations [17]. Another review reported similar results. The survival rates of ART restorations in primary teeth over 2 years were 93% and 62% for single- and multi-surface restorations, respectively [18]. A review found no significant difference between the survival rates of single-surface amalgam and ART restorations in primary teeth over two years (relative risk (RR) 1.07 with 95% confident interval (CI): 0.91–1.27) [19]. Another review found the success rates of ART in restoring occluso-proximal lesions in primary teeth were similar to those of amalgam or composite [20]. Furthermore, a systematic review of different restorative approaches on primary teeth supported the use of ART in young children [21]. Table 1 is a summary of several systematic reviews of ART. These reviews demonstrated evidence that ART is effective and beneficial towards managing ECC in children. A study reported that children receiving ART restorations had lower levels of anxiety when compared with those receiving conventional restorations using electrical drilling [22]. Another study found that the level of anxiety of preschool children towards dental treatment had decreased after ART treatment [23]. Another study also found that ART has good acceptability among young children [16]. Most children (93%) reported no tooth pain during the ART procedure, and 86% were satisfactory to receive ART in the next visit [16].

The experts of the International Caries Consensus Collaboration meeting in 2016 supported the concept of ART [24]. They concluded that a minimally invasive approach should be promoted, in addition to preserving the tooth structure and postponing the cycle of dental fillings. Carious tissue should be excavated just to promote conditions for long-lasting restorations. ART can be an important component in community dental health services for preschool children because of its effectiveness and feasibility [21]. Even though ART has been demonstrated to be favourably acceptable to preschool children and an effective caries management for them beyond the traditional clinical setting, the retention rate for proximal restorations in anterior teeth is low. This either suggests the ART should be modified for such circumstances or that other alternatives such as caries arresting treatment with topical silver or fluoride application should be further studied [16]. This is important, especially in the context of preschool children in China, because their primary incisors are the most caries-affected teeth [25]. The “Assessment of Multiple Systematic Reviews” (AMSTAR) tool (Bruyère Research Institute, Ottawa, ON, Canada) was used to assess the quality of systematic reviews or meta-analyses in Table 1 [26].

Although ART has promising results in treating ECC, it still requires a certain amount of time from trained dentists. In addition, ART is not satisfactory for restoring proximal caries in anterior primary teeth. Due to the very large population in China with a heavy burden of ECC, the development of ART program for Chinese preschool children may face some barriers regarding limited manpower and inadequate supply of materials.

## 3. Caries Management with Fluorides

The progression of tooth decay in children can lead to dental pain, infection and loss of functioning. Caries management by means of less invasive and more cost-effective approaches is an imperative issue. Caries arresting treatment aiming to slow down caries progression, is an alternative and practical solution to reduce children’s discomfort, as well as any consequences due to caries. Studies have shown that enamel caries can be inactivated and remineralised by topical fluorides [28]. When enamel caries progress substantially into dentine, progression rate of dentine caries is more rapid than that of enamel caries [29]. There is demineralisation of the inorganic component and collagen breakdown of the organic component. The recovery of structural integrity is not possible. However, caries lesions can be arrested at any stage during the caries process. Arrested dentine caries are defined by the surface hardness of the dentine, with a yellow to dark brown appearance [29]. Although dental caries causes mineral demineralisation and collagen fibre degradation (affected dentine), the inner layer (affected dentine) is scarcely infected by bacteria. The affected dentine can be remineralised because it still contains reasonably high concentrations of mineral salts. Pulp can remain vital with deep dentine caries without pulp exposure. Caries is arrested when the dentinal tubules in the affected area are deposited with mineral crystals. A histopathological study showed that primary teeth with arrested caries had a significantly better pulpal status than those with active caries [30]. It is possible to monitor arrested cavitated dentine caries without placing a restoration.

The use of topical fluorides can be a useful strategy to arrest ECC. There is evidence that various forms of topical fluorides are effective in preventing and controlling dental caries [28]. Fluoride exhibits anti-caries properties in different ways. The proposed mechanism of fluoride can be explained as follows; the primary preventive effect is a topical effect on erupted teeth [31]. Fluoride concentrated in saliva and dental plaque can inhibit demineralisation of tooth substrate. Fluoride incorporated with calcium and phosphate, which is taken up by demineralised tooth substrate, forms a fluroapatite crystalline structure by remineralisation, thus increasing resistance to the next acid challenge [32]. In addition, fluoride can inhibit the caries process by providing antimicrobial actions such as lowering acid production and limiting glucan synthesis [33]. However, it should be noted that the antibacterial effect occurs at higher levels of fluoride. The implications of antimicrobial actions of low-level fluoride in vivo are still not clear. There are three main modes of delivery of fluorides: community-based fluoridation, self-applied and professionally applied topical fluorides.

### 3.1. Water Fluoridation in Mainland China and Hong Kong

The paramount benefit from fluoride can be achieved by a continuous supply of fluoride at low concentrations for remineralisation. Water fluoridation is the most effective and economical strategy if there is a good water supply system available with rigorous surveillance [34]. It has been proposed that the most influential effect of water fluoridation is not so much in preventing new caries lesions, but in enhancing the remineralisation process of carious lesions and thus arresting the caries process. In Hong Kong, water fluoridation has been successfully implemented as a dental public health measure. The project was established in the 1960s, and has significantly reduced the prevalence of dental caries in young children from 84% in 1968 [35] to 55% in 2017 [25]. In Mainland China, water fluoridation was introduced in 1965 in Guangzhou, located in Southern China, but it was ceased in 1983. The main reason for discontinuing water fluoridation was the endemic dental fluorosis reported in 29 of the 34 provinces in the fluoridated areas [36]. These reports of a high prevalence of tooth fluorosis induced the government administration to stop water fluoridation in all provinces of Mainland China. Thus, regular surveillance of fluoride intake and fluorosis is required to monitor any changes that possibly occur after the implementation.

### 3.2. Self-Applied Fluorides

Topical fluorides can be divided based on their mode of administration, either self-applied or professionally applied. Self-applied topical fluorides eliminate the cost of hiring dental professionals, but not the cost of the fluoride agents, which are frequently consumed in low concentrations. The most commonly used fluoridated products are fluoride toothpastes and fluoride mouth rinses. Sodium fluoride, stannous fluoride or acidulated phosphate fluoride are common active ingredients in fluoride mouth rinses. Fluoride mouth rinse solutions available at 0.05% and 0.2% are for daily use and weekly use, respectively. However, fluoride mouth rinse is generally not recommended for preschool children because they may unintentionally swallow during rinsing, and this poses an increased risk for dental fluorosis in the future.

#### Fluoride Toothpastes

A systematic review confirmed the benefits of using fluoridated toothpaste in preventing dental caries in primary teeth [37]. However, the cost of toothpaste can be an issue in some developing countries. A 3-year community-based trial that studied the effect of tooth brushing with fluoride toothpaste was performed in China [38]. The trial investigated the incidence of the re-hardening of dentine caries in preschool children and found that 45% of the proximal and 23% of the buccal and palatal carious lesions in anterior teeth were inactive. The net caries reduction was 43%. The study concluded that daily tooth brushing with fluoride toothpaste in preschool children could be adopted as an effective school-based programme to control ECC in China. A systematic review using random effects meta-analyses on 74 included studies found that the effect of fluoridated toothpaste increased significantly with higher caries experience at baseline, higher concentration of fluoride and more frequent use and supervised toothbrushing [39]. Unfortunately, some child toothpastes do not contain fluorides in China. Use of fluoridated toothpaste in China, particularly in remote areas, can be hampered by its poor logistic and distribution as well as its relatively high cost [34]. This is a challenge for the development of the fluoridated toothpaste market in Mainland China, both to product manufacturers and to dental health professionals.

### 3.3. Professionally Applied Fluorides

There are various types of professionally applied fluoride agents. The three commonly used forms are fluoride gel, varnish and solution. The decision to choose any fluoride agents should be based on both scientific evidence and practical considerations. More importantly, the fluoride product must be proven to be effective for caries prevention.

#### 3.3.1. Fluoride Gels

Professionally applied fluoride gels usually contain 2% sodium fluoride or 1.23% acidulated phosphate fluoride. A review suggested that the application of fluoride gels, either professionally or self-applied, is associated with a great reduction in caries increment [40]. Moreover, the review reported that the effect was independent of other fluoride sources but did depend on application frequency. Most fluoride gels are flavoured to enhance children’s acceptance, and are applied in a mouth tray for ease of clinical application. Acidulated phosphate fluoride gel was considered the most popular professionally applied fluoride in the 1980s. However, fluoride solutions and gels have some disadvantages due to the increased risk of over-ingestion, particularly in young children. Furthermore, substantial leaching of absorbed fluoride from the enamel surface occurs within the first 24 h after fluoride gel or solution application [33].

#### 3.3.2. Sodium Fluoride Varnishes

Fluoride varnishes can adhere to tooth surfaces for longer periods of time and prevent the immediate loss of fluoride after topical application, thus acting as slow-releasing reservoirs of fluoride. Fluoride varnishes usually contain 5% sodium fluoride (22,600 ppm fluoride). Several trials have shown fluoride varnish to be effective in caries prevention, with an average reduction of about 30% [33]. Milgrom and his co-workers also reported that regular application of 5% sodium fluoride varnish could arrest caries [41]. However, another study reported that the application of fluoride varnish every three months is not effective to arrest active dentine caries in children [42].

#### 3.3.3. Silver Diamine Fluoride Solutions

In China and Japan, silver diamine fluoride (SDF) has been adopted for arresting ECC in children for many years [34]. The use of SDF is currently gaining more popularity due to the favourable results of arresting dentine caries in primary teeth [42,43,44,45]. SDF was accepted as a therapeutic agent to treat caries in Japan more than 40 years ago [31]. Studies investigating the caries arrest effect have been conducted in preschool children in China [42]. A randomized clinical trial in Chinese preschool children confirmed that 38% SDF was effective in arresting dental caries in primary teeth. No extra benefit was found when removing soft caries before SDF or sodium fluoride application [42]. Another trial carried out in China compared the effectiveness of glass ionomer cement restoration and SDF. The study concluded that no differences existed in the arresting effect of 38% SDF applied annually and the restoration with glass ionomer cement in primary teeth over 2 years [45]. More recently, a clinical trial conducted in Hong Kong found that 3 applications with weekly intervals of SDF is as effective as an annual application of SDF in arresting dentine caries after 18 months [44]. Different concentrations of SDF (12% vs. 38%) and different periodicities of SDF application (annual vs. semi-annual) were investigated. It was shown that 38% SDF showed a more beneficial effect than 12% SDF did when applied semi-annually rather than annually [43]. Caries arrest rates of 12% SDF annually, 12% SDF semi-annually, 38% SDF annually and 38% SDF semi-annually were 55%, 59%, 67% and 76%, respectively (*p* < 0.001) [43]. Table 2 summarises the randomised clinical trials of SDF conducted in young children in China. It can be a simple, non-invasive and low-cost treatment. However, SDF stains caries black, and the children and their parents must be well informed before application.

It should be noted that this is a critical review to discuss updated dental public health measures and their effectiveness in preventing and managing ECC in the context of contemporary China. Due to a wide range of study types from epidemiological studies to systematic reviews included, only some key included studies in this review were critically appraised. In Table 2, the Cochrane Collaboration’s tool [49] was adopted to assess the risk of bias of the included studies. Six aspects, including selection bias, performance bias, detection bias, attrition bias, reporting bias and other bias, were assessed, accordingly. The quality of the included SDF clinical trials was graded by the assessment tool “Quality Assessment of Controlled Intervention Study” [50]. Three clinical trials [43,44,45] were evaluated as having low risk of bias and good quality.

Although there were some SDF trials in Hong Kong and Mainland China with promising results, the adoption of SDF in Mainland China has been still limited. To date, there is no approval from China Food and Drug administration, and no Chinese SDF commercial brands are available in Mainland China [51,52]. This calls for action by the National Health and Family Planning Commission of the People’s Republic of China in order to clear SDF for market in Mainland China.

## 4. Conclusions

The prevalence and severity of caries among preschool children are high in China. The inadequate number of dentists and the high cost of traditional restorative treatment make it impractical to manage ECC using conventional methods. Promoting use of topical fluorides at a population level is required. School-based tooth brushing with fluoride toothpaste has been demonstrated with some success, and may be used to arrest ECC. However, this strategy alone may not be capable of dealing with the high prevalence of active caries in Chinese preschool children. ART has promising results in treating ECC in a community setting. However, due to a large number of caries-affected preschool children and limited resources in China, topical fluoride, particularly SDF, may be a better alternative for caries control due to its cost-effectiveness, safe and practicability to implement. Its use as a community measure for treating established caries in children is worth investigation.

## Figures and Tables

**Table 1:** Systematic reviews on the effectiveness of atraumatic restorative treatment on primary teeth.

Authors, Year [Reference]	No. of Studies Included	Main Findings	Quality Assessment *
Van’t Hof et al. 2006 [17]	28	The 3-year survival rate for single-surface ART restorations was 86% Higher annual failure rate (17%) of multi-surface ART restorations, compared to that (5%) of single-surface restorations	Critically low
Mickenautsch et al. 2010 [19]	7	No difference in the 2-year retention rates of ART and amalgam for single-surface restorations	Moderate
de Amorim et al. 2012 [18]	29	The respective 2-year survival rates of single- and multi-surface ART restorations were 93% and 62%	Low
Duangthip et al. 2016 [21]	9	The use of less-invasive approach with ART is beneficial in managing ECC in young children	Moderate
Tedesco et al. 2017 [27]	4	No difference in survival rate between ART and conventional Class II restorations	Moderate

* The quality assessment was based on the “Assessment of Multiple Systematic Reviews” (AMSTAR) tool [26].

**Table 2:** Clinical trials of silver diamine fluoride (SDF) to arrest caries in young children in China.

Author, Year [Reference]	Duration, Children	Intervention Group	Caries Arrest Rate	Risk of Bias */Quality **
Wang et al. 1984 [46]	18 months, 68 children, 3.5–4.5 years	Gp 1: 38% SDF every 3 to 4 months Gp 2: Control	Gp 1: 85% Gp 2: 31% (*p* < 0.001)	Moderate/Poor
Ye et al. 1995 [47]	12 months, 300 primary teeth	38% SDF, one off	92%	High/Poor
Chu et al. 2002 [42]	30 months, 375 children, 3–5 years	Gp 1: Caries removal + 38% SDF once yearly Gp 2: 38% SDF once yearly Gp 3: Caries removal + 5% NaF 4 times yearly Gp 4: 5%NaF 4 times yearly Gp 5: Water (Control)	Gp 1: 60% Gp 2: 66% Gp 3: 37% Gp 4: 40% Gp 5: 34% (*p* < 0.001)	Moderate/Fair
Yang et al. 2002 [48]	6 months, 50 children, 3–6 years	38% SDF, one off	94%	High/Poor
Zhi et al. 2012 [45]	24 months, 212 children, 3–4 years	Gp 1: 38% SDF once yearly Gp 2: 38% SDF 2 times yearly	Gp 1: 79%, Gp 2: 91% (*p* < 0.05)	Low/Good
Duangthip et al. 2016 [44]	18 months, 304 children, 3–4 years	Gp 1: 30% SDF once yearly Gp 2: 30% SDF 3 times weekly at baseline Gp 3: 5% NaF 3 times weekly at baseline	Gp 1: 40% Gp 2: 35% Gp 3: 27% (*p* < 0.001)	Low/Good
Fung et al. 2017 [43]	30 months, 888 children, 3–4 years	Gp 1: 12% SDF once yearly Gp 2: 12% SDF 2 times yearly Gp 3: 38% SDF once yearly Gp 4: 38% SDF 2 times yearly	Gp 1: 55% Gp 2: 59% Gp 3: 67% Gp 4: 76% (*p* < 0.001)	Low/Good

* Risk of bias was assessed by using the Cochrane Collaboration’s tool [49]; ** Quality of study was assessed by using the Assessment of Controlled Intervention Study [50].

## References

[1] American Academy of Pediatric Dentistry (2016). Policy on Early Childhood Caries (ECC): Classifications, Consequences, and Preventive Strategies. Pediatr. Dent..

[2] World Health Organization What is the Burden of Oral Disease? Oral Disease Burdens and Common Risk Factors. http://www.who.int/oral_health/disease_burden/global/en/.

[3] Aggeryd T. (1983). Goals for oral health in the year 2000: Cooperation between WHO, FDI and the national dental associations. Int. Dent. J..

[4] Duangthip D., Gao S.S., Lo E.C.M., Chu C.H. (2017). Early childhood caries among 5- to 6-year-old children in Southeast Asia. Int. Dent. J..

[5] National Health and Family Planning Commission of the People’s Republic of China The Report of the Forth National Oral Health Survey. http://so.nhfpc.gov.cn/.

[6] Qi X. (2008). Report of the Third National Oral Health Survey.

[7] Zhang X., Yang S., Liao Z., Xu L., Li C., Zeng H., Song J., Zhang L. (2016). Prevalence and care index of early childhood caries in mainland China: Evidence from epidemiological surveys during 1987–2013. Sci. Rep..

[8] Wong M.C.M., Lo E.C.M., Schwarz E., Zhang H.G. (2001). Oral health status and oral health behaviors in Chinese Children. J. Dent. Res..

[9] Li Y., Wulaerhan J., Liu Y., Abudureyimu A., Zhao J.B. (2017). Prevalence of severe early childhood caries and associated socioeconomic and behavioral factors in Xinjiang, China: A cross-sectional study. BMC Oral Health.

[10] Sun H.B., Zhang W., Zhou X.B. (2017). Risk Factors associated with Early Childhood Caries. Chin. J. Dent. Res..

[11] Sun X., Bernabé E., Liu X., Gallagher J.E., Zheng S. (2017). Early life factors and dental caries in 5-year-old children in China. J. Dent..

[12] Federation Dentaire Internationale (1991). FDI Basic Facts 1990: Dentistry around the World.

[13] Liu J., Zhang S.S., Zheng S.G., Xu T., Si Y. (2016). Oral Health Status and Oral Health Care Model in China. Chin. J. Dent. Res..

[14] Frencken J.E., Pilot T., Songpaisan Y., Phantumvanit P. (1996). Atraumatic restorative treatment (ART): Rationale, technique, and development. J. Public Health Dent..

[15] Frencken J.E. (2014). The state-of-the-art of ART sealants. Dent. Update.

[16] Lo E.C.M., Holmgren C. (2001). Provision of Atraumatic Restorative Treatment (ART) restorations to Chinese pre-school children—A 30-month evaluation. Int. J. Paediatr. Dent..

[17] Van’t Hof M.A., Frencken J.E., van Palenstein H.W., Holmgren C.J. (2006). The atraumatic restorative treatment (ART) approach for managing dental caries: A meta-analysis. Int. Dent. J..

[18] De Amorim R.G., Leal S.C., Frencken J.E. (2012). Survival of atraumatic restorative treatment (ART) sealants and restorations: A meta-analysis. Clin. Oral Investig..

[19] Mickenautsch S., Yengopal V., Banerjee A. (2010). Atraumatic restorative treatment versus amalgam restoration longevity: A systematic review. Clin. Oral Investig..

[20] Raggio D.P., Hesse D., Lenzi T.L., AB Guglielmi C., Braga M.M. (2013). Is Atraumatic restorative treatment an option for restoring occlusoproximal caries lesions in primary teeth? A systematic review and meta-analysis. Int. J. Paediatr. Dent..

[21] Duangthip D., Jiang M., Chu C.H., Lo E.C.M. (2016). Restorative approaches to treat dentin caries in preschool children: Systematic review. Eur. J. Paediatr. Dent..

[22] Schriks M., Van Amerongen W. (2003). Atraumatic perspectives of ART: Psychological and physiological aspects of treatment with and without rotary instruments. Community Dent. Oral Epidemiol..

[23] Ishan K.K., Agarwal V., Gupta B.D., Anand R., Sharma A., Kushwaha S., Khan K. (2017). Anxiety Levels among Five-Year-Old Children Undergoing ART Restoration- A Cross-Sectional Study. J. Clin. Diagn. Res..

[24] Schwendicke F., Frencken J., Bjørndal L., Maltz M., Manton D., Ricketts D., Van Landuyt K., Banerjee A., Campus G., Doméjean S. (2016). Managing carious lesions: Consensus recommendations on carious tissue removal. Adv. Dent. Res..

[25] Chen K.J., Gao S.S., Duangthip D., Li S.K.Y., Lo E.C.M., Chu C.H. (2017). Dental caries status and its associated factors among 5-year-old Hong Kong children: A cross-sectional study. BMC Oral Health.

[26] Shea B.J., Reeves B.C., Wells G., Thuku M., Hamel C., Moran J., Moher D., Tugwell P., Welch V., Kristjansson E. (2017). AMSTAR 2: A critical appraisal tool for systematic reviews that include randomised or non-randomised studies of healthcare interventions, or both. BMJ.

[27] Tedesco T.K., Calvo A.F., Lenzi T.L., Hesse D., Guglielmi C.A., Camargo L.B., Gimenez T., Braga M.M., Raggio D.P. (2017). ART is an alternative for restoring occlusoproximal cavities in primary teeth-evidence from an updated systematic review and meta-analysis. Int. J. Paediatr. Dent..

[28] Gao S.S., Zhang S., Mei M.L., Lo E.C.M., Chu C.H. (2016). Caries remineralisation and arresting effect in children by professionally applied fluoride treatment—A systematic review. BMC Oral Health.

[29] Chu C.H., Lo E.C.M. (2008). Microhardness of dentine in primary teeth after topical fluoride applications. J. Dent..

[30] Di Nicolo R., Guedes-Pinto A.C., Carvalho Y.R. (2000). Histopathology of the pulp of primary molars with active and arrested dentinal caries. J. Clin. Pediatr. Dent..

[31] Chu C.H., Lo E.C.M. (2008). Promoting caries arrest in children with silver diamine fluoride: A review. Oral Health Prev. Dent..

[32] Mei M.L., Nudelman F., Marzec B., Walker J.M., Lo E.C.M., Walls A.W., Chu C.H. (2017). Formation of Fluorohydroxyapatite with Silver Diamine Fluoride. J. Dent. Res..

[33] Chu C.H., Mei M.L., Lo E.C.M. (2010). Use of fluorides in dental caries management. Gen. Dent..

[34] Burt B.A., Eklund S.A. (2005). Dentistry, Dental Practice, and the Community.

[35] Wong K.K. (1968). Report of a Dental Survey in Hong Kong 1968.

[36] Wang L.F., Huang J.Z. (1995). Outline of control practice of endemic fluorosis in China. Soc. Sci. Med..

[37] Walsh T., Worthington H.V., Glenny A.M., Appelbe P., Marinho V.C., Shi X. (2010). Fluoride toothpastes of different concentrations for preventing dental caries in children and adolescents. Cochrane Database Syst. Rev..

[38] Lo E.C.M., Schwarz E., Wong M.C.M. (1998). Arresting dentine caries in Chinese preschool children. Int. J. Paediatr. Dent..

[39] Marinho V.C., Higgins J.P., Sheiham A., Logan S. (2003). Fluoride toothpastes for preventing dental caries in children and adolescents. Cochrane Database Syst. Rev..

[40] Weintraub J.A. (2002). Fluoride gel applications reduce caries incidence. Evid. Based Dent..

[41] Milgrom P., Rothen M., Spadafora A., Skaret E. (2001). A case report: Arresting dental caries. J. Dent. Hyg..

[42] Chu C.H., Lo E.C.M., Lin H.C. (2002). Effectiveness of silver diamine fluoride and sodium fluoride varnish in arresting dentin caries in Chinese pre-school children. J. Dent. Res..

[43] Fung M.H.T., Duangthip D., Wong M.C.M., Lo E.C.M., Chu C.H. (2017). Randomized Clinical Trial of 12% and 38% Silver Diamine Fluoride Treatment. J. Dent. Res..

[44] Duangthip D., Chu C.H., Lo E.C.M. (2016). A randomized clinical trial on arresting dentine caries in preschool children by topical fluorides—18 month results. J. Dent..

[45] Zhi Q.H., Lo E.C.M., Lin H.C. (2012). Randomized clinical trial on effectiveness of silver diamine fluoride and glass ionomer in arresting dentine caries in preschool children. J. Dent..

[46] Wang S. (1984). Clinical observation of silver diamine fluoride in arresting dental caries. J. Cap. Med. Univ..

[47] Ye Z. (1995). The use of 38% silver diamine fluoride in dental caries. Chin. J. Conserv..

[48] Yang Q., Wei B., Ye Z. (2002). Clinical effectiveness of using silver diamine fluoride to treat caries on primary anterior teeth. Heilongjiang Med. Pharmacol..

[49] The Cochrane Collaboration (2011). Cochrane Handbook for Systematic Reviews of Interventions Version 5.1.0.

[50] U.S. Department of Health & Human Services Quality Assessment of Controlled Intervention Study. https://www.nhlbi.nih.gov/health-topics/study-quality-assessment-tools.

[51] China Food and Drug Administration. http://www.sda.gov.cn/WS01/CL0412/.

[52] Chinese Marketed Drugs Database. http://www.drugfuture.com/cndrug/#.

